# Cardiac Multimodality Imaging in Hypertrophic Cardiomyopathy: What to Look for and When to Image

**DOI:** 10.2174/1573403X19666230316103117

**Published:** 2023-07-17

**Authors:** Perry Wengrofsky, Yonatan Akivis, Inna Bukharovich

**Affiliations:** 1 Division of Cardiology, Department of Medicine, Rutgers New Jersey Medical School, Newark, NJ 07103, USA;; 2 Department of Medicine, SUNY Downstate Health Sciences University, Brooklyn, NY 11203, USA;; 3 Division of Cardiology, Department of Medicine, NYC Health and & Hospitals, Kings County, Brooklyn, NY 11203, USA

**Keywords:** Hypertrophic cardiomyopathy, multimodality imaging, echocardiography, cardiac magnetic resonance imaging, cardiac computed tomography, cardiac death risk

## Abstract

Hypertrophic cardiomyopathy (HCM), now recognized as a common cardiomyopathy of complex genomics and pathophysiology, is defined by the presence of left ventricular hypertrophy of various morphologies and severity, significant hemodynamic consequences, and diverse phenotypic, both structural and clinical, profiles. Advancements in cardiac multimodality imaging, including echocardiography, cardiac magnetic resonance imaging, and cardiac computed tomography, with and without angiography have greatly improved the diagnosis of HCM, and enable precise measurements of cardiac mass, volume, wall thickness, function, and physiology. Multimodality imaging provides comprehensive and complementary information and hasemerged as the bedrock for the diagnosis, clinical assessment, serial monitoring, and sudden cardiac death risk stratification of patients with HCM. This review highlights the role of cardiac multimodality imaging in the modern diagnosis and management of HCM.

## 1 INTRODUCTION

Since the late 1950s’ and early 1960s’ clinical discoveries and hemodynamic descriptions of hypertrophic cardiomyopathy (HCM, originally called “asymmetrical hypertrophy” by Torre and “idiopathic hypertrophic subaortic stenosis” by Brockenbrough, Braunwald, and Morrow, the understanding of the prevalence, pathophysiology, and manifestations of HCM has greatly increased [1, 2]. HCM, once considered a rare pathology, is now recognized as widely distributed with a spectrum of phenotypes, ranging from incidental findings on testing in asymptomatic patients to progressive symptoms related to dynamic left ventricular outflow tract (LVOT) obstruction, mitral regurgitation (MR), tachyarrhythmia, nonobstructive heart failure, or sudden cardiac death (SCD) [3].

At the heart of the diagnosis, risk stratification, and longitudinal care of HCM patients, both symptomatic and asymptomatic, is multimodality imaging with echocardiography (ECHO), cardiac magnetic resonance imaging (CMR), cardiac computed tomography (CCT), with and without angiography [4].

Multimodality imaging enables the characterization of the diverse morphologic expression of HCM and the identification of established clinical risk factors for SCD as outlined in the 2020 American Heart Association/American College of Cardiology (AHA/ACC) HCM guidelines [5]. Building on the 2020 guidelines, the American Society of Echocardiography/American Society for Nuclear Cardiology/Society for Cardiovascular Magnetic Resonance/Society for Cardiovascular Computed Tomography (ASE/ASNC/ SCMR/SCCT) in 2022, has recently published recommendations for the utilization of multimodality imaging in the care of HCM patients [6]. An appropriate understanding of the capabilities of existing and emerging imaging techniques is critical for every cardiologist, from the non-invasive to the advanced imager, in the care of HCM patients and their families [4, 6].

In this review, we will focus on the role of cardiac multimodality imaging, particularly echocardiography, CMR, and CCT, in the diagnosis and management of HCM, and highlight the strengths and applications of each modality in the identification, screening, risk stratification, and surveillance monitoring of this common, globally distributed, inheritable, yet incredibly diverse cardiac pathology.

## 2 PHENOTYPES, GENOTYPES, AND CLINICAL PATHOPHYSIOLOGY

According to the 2020 AHA/ACC guidelines, HCM is defined by hypertrophy of the left ventricle (LV) with a maximal end-diastolic wall thickness ≥15 mm anywhere in the LV, in the absence of another cardiac, systemic, metabolic, or syndromic disorders that would cause LV hypertrophy (LVH), such as aortic stenosis, hypertension, or amyloidosis [5, 7]. Mild hypertrophy (13-14 mm) can be diagnostic of HCM when present in a family member of an HCM patient, or in combination with positive genetic testing [5]. Systemic hypertension is a specific comorbidity that commonly cooccurs with HCM, contributes to LVH, and can complicate the diagnosis of HCM in patients with an LV wall thickness <20 mm, but particular structural patterns will point more to HCM [3].

The LVH can be symmetric and global, but is typically asymmetric, most commonly involving the basal interventricular septum below the aortic valve [8]. Multiple distinct morphologic subtypes of HCM exist with patterns of localized regional hypertrophy, including reverse septal curvature, apical, and mid-cavitary with apical aneurysm [9]. Symptomology and clinical profiles are related to the pattern of hypertrophy, with the syndrome of hypertrophic obstructive cardiomyopathy (HOCM) classically arising from basal septal LVOT obstruction (LVOTO) and less commonly from midventricular obstruction, and other nonobstructive symptoms such as heart failure with preserved ejection fraction and atrial fibrillation patterns stemming from decreased LV compliance, elevated filling pressures, and left atrial (LA) dilation [3]. SCD, the rarest and most devastating complication of HCM, is associated with specific a high-risk structural and electrophysiologic findings and can occur in previously asymptomatic patients or be the initial manifestation of HCM [10].

HCM has been identified in 125 countries, carrying an estimated prevalence of 1:200-1:500, with a plurality (46%) of patients having asymptomatic and benign clinical profiles. Among symptomatic patients, 43% have progressive HF symptoms (39% with obstructive physiology, 4% with non-obstructive physiology), and 6% have ventricular tachyarrhythmia (malignant and non-malignant), with an annual incidence of < 1% of SCD among adult HCM patients [3, 10]. Despite the low incidence and longitudinal risk of SCD, HCM has been identified as the cause of sudden death in 36% of competitive athletes [3].

Beyond symptomology, HCM is characterized as an autosomal dominant genetic disorder with variable penetrance and phenotypic expression due to a confluence of genetic and non-genetic factors [8]. A total of 60% of HCM patients have a recognizable familial transmission pattern, with population and familial genetic analysis identifying culprit mutations in sarcomeric proteins, sarcomere-associated proteins, and Z-disk proteins [8, 11]. In all, 5% of HCM patients have digenic or oligogenic causal mutations in one or multiple genes, and in sporadic small familial clusters, there may be a “missing causal gene” due to difficulty in identifying the culprit mutation [8].

Genetic mutations in sarcomeric and associated proteins cause multiple cellular changes that collectively result in micro- and macroscopic abnormalities, primarily abnormal myofibril structure and arrangement, coronary artery microvascular dysfunction, and patchy yet widely distributed micro-scarring and perivascular fibrosis [11-13]. These alterations arise from diverse hypothetical pathogenic pathways, including increased calcium affinity, inefficient contractility, myocardial energy deficiency, reactive oxygen species production, harmful protein accumulation, and proliferation of non-cardiomyocyte cells like fibroblasts [11, 14].

The pathophysiology of HCM is diverse and multifaceted, and the clinical symptoms and outcomes for a given patient may be dominated by one specific component or a complex interplay of multiple components. LVOTO is present in nearly ¾ of HCM patients, can be present at rest or exertion (and worsened by exertional provocation), and is caused by abnormal flow from the anatomical narrowing of the LVOT [5]. During systolic, narrowing arises from the thickening of an already asymmetrically hypertrophied basal septum and dynamic displacement of the mitral valve (MV) leaflets anteriorly (systolic anterior motion of mitral valve, SAM) that themselves may be abnormal, with alterations that can include longer leaflets and anterior papillary muscle displacement [5, 15].

LVOTO and SAM cause both high intracavitary pressures, further begetting LVH, and MR from the loss of leaflet coaptation [5]. LVH causes diastolic dysfunction through decreased chamber compliance, resulting in high intracavitary filling pressures [5, 16]. Myocardial ischemia in HCM can be present even without epicardial coronary atherosclerotic obstructive disease and is mainly attributable to myocardial oxygen supply-demand mismatch secondary to myocardial hypertrophy, microvascular dysfunction, and impaired coronary flow reserve, all of which are worsened by the increased wall stress from high intracavitary pressures [5, 17].

A thorough comprehension of HCM pathophysiology is fundamental to the evaluation of suspected or confirmed HCM, and when complemented with the appropriate knowledge of the capabilities of existing and novel cardiac multimodality imaging techniques, enables the proper selection of targeted imaging on a personalized and individualized patient basis, guiding pharmacologic, surgical, and electrophysiologic interventions, as well as long term monitoring and reassessment with serial reimaging as outlined in national and international guidelines.

## 3 ECHOCARDIOGRAPHY

### 3.1 General Considerations and Guidelines

Transthoracic echocardiography (TTE) is the initial imaging modality of choice in patients with confirmed or suspected HCM, and while historically 2-dimensional (2D) echocardiography, M-mode, and color Doppler echocardiography have been key to the diagnosis of HCM, the evolution and incorporation of newer ultrasound-based technologies like tissue Doppler, speckle tracking and strain analysis, 3-dimensional (3D) echocardiography, and ultrasound enhancing agents (UEAs) have improved and expanded the echocardiographic assessment of HCM [6, 18]. These novel technologies provide important information on differences in regional contractility and myocardial mechanics, and diastolic function for the pre-clinical diagnosis and risk stratification in both patients and their relatives [18-20].

According to the 2020 ACC/AHA guidelines, in patients with HCM who have undergone initial TTE assessment, repeat TTE is recommended every 1 to 2 years in patients with no change in clinical status or adverse events to serially reassess the degree and pattern of LVH, dynamic LVOTO, and myocardial function, but should be repeated as soon as possible if there is a change in clinical status or one suffers a new clinical event. Screening echocardiography and serial reimaging is also recommended in asymptomatic family members of HCM patients determined to be at risk for developing HCM based on family history and genotype status and can be considered in more distant relatives based on clinical judgement. For asymptomatic adults, TTE screening should be initiated at the time of HCM diagnosis in a first-degree relative and should be repeated every 3 to 5 years, or sooner, depending on the development of symptoms or familial history of early malignant HCM phenotypic courses [5].

While TTE is the initial non-invasive imaging modality of choice, transesophageal echocardiography (TEE) should be considered when TTE findings are inconclusive, especially when findings can have significant ramifications for management, including the planning for surgical reduction therapy, advanced assessment of MV anatomy and determination of etiology of MR, and exclusion of subaortic membranes that can masquerade as an asymmetric basal septal thickening and contribute to LVOTO [5].

**Fig. (1):**
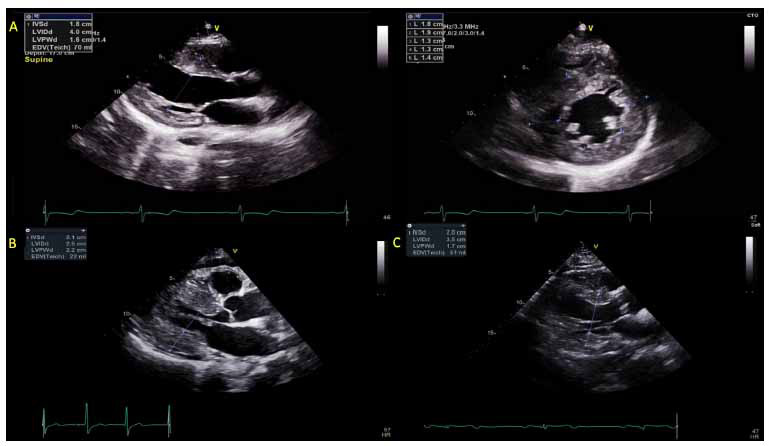
TTE images representing patterns and severity of LVH in the phenotypic expressions of HCM. (**A**) (row) = parasternal long (top left) and short (right) axis views of a patient with concentric/neutral HCM. (**B** and **C**) = parasternal long axis views of more severe hypertrophy in two patients with HOCM.

Fundamental to the echocardiographic of HCM is the accurate quantification of location, pattern, and degree of LVH (Fig. **1**), with end-diastolic wall thickness ≥15 mm (or ≥ 13 mm in an individual with a known disease-causing genetic mutation or a family history of HCM) being diagnostic of HCM [5, 6].

HCM exists in different phenotypic expressions that are usually asymmetric, affect regional and non-contiguous LV segments, and may involve the right ventricle (RV) which can globally or regionally hypertrophy (RVH) in a similarly heterogeneous fashion [6]. While asymmetric basal septal hypertrophy (with or without dynamic LVOTO) is the most common HCM phenotype, representing nearly 37%-50% of HCM patients, multiple phenotypic variants exist, including the sigmoidal septum, reversed septal curvature, concentric, mid-wall, and apical [21-23]. Among the non-basal septal HCM morphologies, the sigmoidal septum is the most common with a prevalence of 40-50% [22].

Optimal TTE characterization of pattern and degree of hypertrophy requires avoidance of LV foreshortening in the apical 4-, 3-, and 2-chamber views, particularly in cases of suspected apical HCM, and proper discernment of LV and RV structures in the parasternal long axis views, such as papillary muscles and moderator band that can cause an overestimation of wall thickness [6, 24]. RVH, defined as an increased RV wall thickness of ≥ 8 mm), has a prevalence of over 33% in patients with HCM, and should be assessed in the subcostal views at end-diastole to avoid overestimation by the inclusion of epicardial fat [6, 24].

High-quality 3D-echocardiography can provide advanced mechanical insights beyond the anatomical and functional analysis of the LV, including identification of focally hypertrophied areas, location and extent of LV cavity obliteration, better delineation of possible apical thrombi, and deformational geometry of the LVOT [6, 25]. On both TTE and TEE, 3D-echocardiography provides greater anatomical and physiological information on the mitral valve apparatus and can help elucidate mechanisms of MR, LVOTO, and SAM, including papillary muscle hypertrophy or anterior displacement, mitral leaflet elongation, and mitral coaptation point displacement [25-28].

**Fig. (2):**
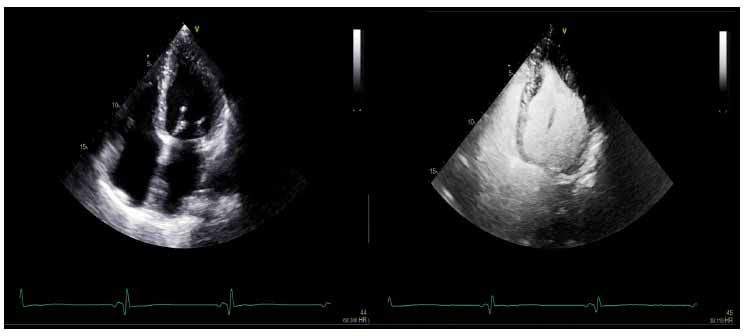
Apical 4 chamber TTE view without (left) and with (right) contrast enhancement, providing a better definition to asymmetric apical hypertrophy in a patient with apical HCM.

While 3D echocardiography can help evaluate for LV crypts and thrombi, contrast echocardiography (Fig. **2**) with the utilization of UEAs improves the identification of apical aneurysm and enhances delineation of endocardial borders for better quantification of LV wall thickness [6, 29, 30].

#### 3.1.1 Left Ventricular Systolic Function

**Fig. (3):**
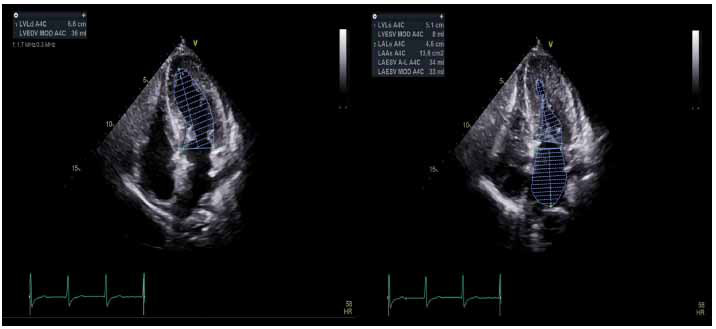
Quantitative assessment of LVEF in the patient with HOCM, demonstrating hyperdynamic systolic function with LVEF of 77%. Using the Simpsons biplane method, LVEF is obtained by averaging measurements in the apical 4-chambers (shown) and apical 2-chamber views.

The 2020 ACC/AHA HCM and 2022 ASE/ASNC/ SCMR/SCCT guidelines and recommendations define an LV ejection fraction (LVEF), determined with a high-quality 2D or 3D-echocardiography, with or without the use of UEAs, of 50% as the lower limit of normal in HCM, and systolic dysfunction with an LVEF < 50% has been repeatedly shown to be associated with increased all-cause mortality and risk of SCD, as well as higher rates of cardiac transplantation and need for LV assist device implantation [5, 6]. The overwhelming majority of HCM patients have normal to hyperdynamic LV systolic function (Fig. **3**), with systolic dysfunction reported in 5-10% of HCM cohorts [6]. The 2D echocardiographic assessment of LVEF with quantitative biplane techniques remains the predominant modality for the initial and serial evaluation, with considerable inter-observer variability that can be mitigated with the use of UEAs and 3D-echocardiography [6, 31].

**Fig. (4):**
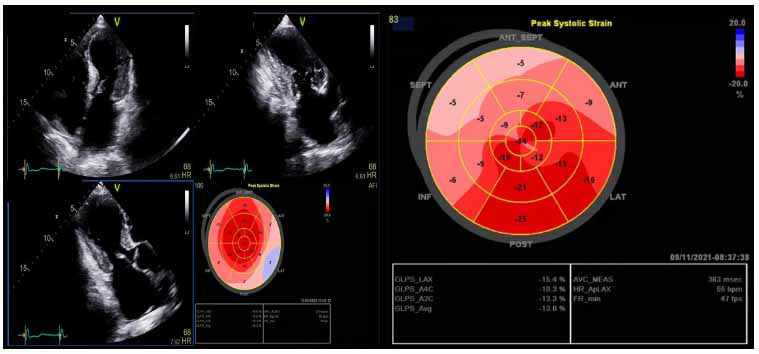
Patterns of regional and global strain in HOCM.

**Fig. (5):**
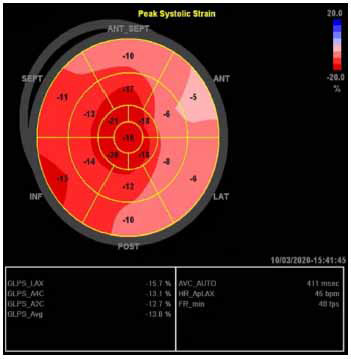
Pattern of regional and global strain in a non-HOCM variant (concentric/neutral).

Speckle tracking and systolic strain analysis, both qualitative and quantitative with LV global longitudinal strain (LV-GLS), enables the identification of subclinical systolic dysfunction in patients with normal LVEF and preserved global and regional wall motion [6]. LV-GLS patterns, both regionally and globally, can vary greatly, with abnormalities typically localizing to regions of hypertrophy [6, 32, 33]. LV-GLS rates are lower in HCM (Figs. **4** and **5**) than in normal healthy controls, and across HCM populations, abnormal GLS is associated with adverse composite cardiac outcomes, ventricular tachyarrhythmia, and can be abnormal even in the absence of hypertrophy in HCM mutation carriers [20, 34].

#### 3.1.2 Left Ventricular Diastolic Function

Diastolic dysfunction in HCM is a major contributor to symptoms in those with and without LVOT of other cavitary obstruction and results from a combination of impaired LV compliance and relaxation and associated elevated filling pressures, as well as atrial myopathy and further impairment of LV diastolic filling [5, 6, 16, 35]. The 2020 ACC/AHA and 2022 ASE/ASNC/SCMR/SCCT guidelines recommend that the echocardiographic evaluation of the HCM includes a comprehensive assessment of diastolic function, which includes mitral inflow velocities, early diastolic velocity by tissue Doppler, peak tricuspid regurgitation (TR) velocity (TR Vmax), biplane LA volume with LA volume indexing (LAVI) to body surface area, and pulmonary vein flow [5, 6].

**Fig. (6):**
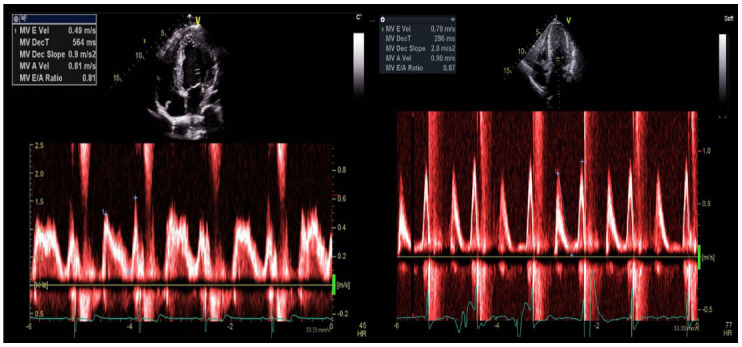
Mitral inflow velocity measured at leaflet tips for a young patient with non-HOCM variant (left) and elderly patient with HOCM (right).

Mitral inflow velocities (Fig. **6**), namely early (E) passive and late/atrial (A) active filling velocities and the resultant E/A ratio, can vary in HCM, with restrictive LV filling pattern (E/A > 2.0, with or without E > 0.5 m/s) appearing in approximately 6% of HCM patients at initial evaluation [36, 37].

**Fig. (7):**
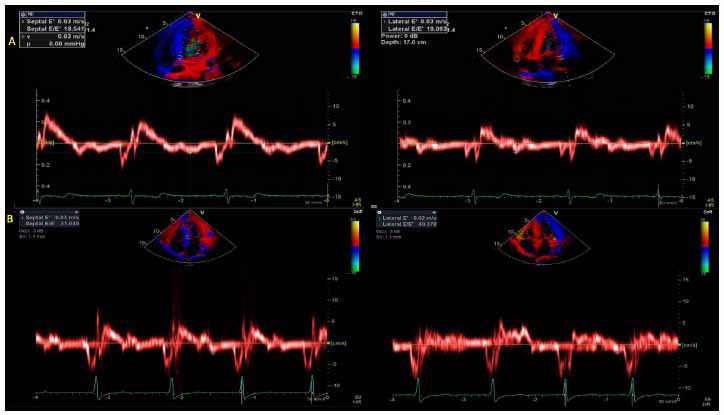
Tissue Doppler velocities measured at the septal and lateral annulus from the same young non-HOCM variant patient (row **A**) and elderly HOCM patient (row **B**), both with increased E/e’ ratio (>14), consistent with elevated mean LA pressure.

E velocity and E/A ratio are among the core variables for the classification and grading of diastolic dysfunction according to the 2016 ASE/EACVI (European Association of Cardiovascular Imaging) diastolic function guidelines, particularly the diagnostic algorithm for assessing diastolic dysfunction in subjects with myocardial disease and both normal and depressed LV EF [38]. Tissue Doppler early diastolic velocity (e’) at the septal aspect and lateral aspect of the mitral annulus (Fig. **7**) sheds light on impaired LV diastolic relaxation, with diminished septal and lateral e’ velocities associated with increased myocardial stiffness in symptomatic and asymptomatic patients with HCM [39, 40]. Elevations in the E/e’ ratio (E/e’ > 14), in addition to LAVI (> 34 mL/m^2^) and TR Vmax (> 2.8 m/s) are utilized to determine LA pressure elevation in line with 2016 ASE/EACVI diastolic function guidelines, with abnormal E/e’ ratio and LAVI being independently associated with adverse outcomes with HCM and providing valuable prognostic information [6, 38-42].

Confounding the assessment of diastolic function and LA pressure are certain pathophysiologic hallmarks of HCM, including MR and atrial fibrillation (AFIB). MR of increasing severity can affect LAVI, E/e’ regardless of underlying LA pressure [6]. In the setting of AFIB, the 2016 ASE/EACVI diastolic function guidelines outline the specific measurements that suggest elevated LV filling and LA pressures, particularly a short mitral deceleration time ≤ 160 msec [38]. Other variables, including peak acceleration rate of mitral E velocity, isovolumetric relaxation time, and pulmonary venous diastolic deceleration time velocity are not as well studied in HCM patients with AFIB [6, 38]. Whereas GLS is well studied in HCM, there is presently limited but emerging data on the application of speckle tracking and strain analysis for diastolic dysfunction, including global diastolic strain and LA strain [6, 43-47].

#### 3.1.3 Left Ventricular Outflow Tract Obstruction and Mitral Regurgitation

**Fig. (8):**
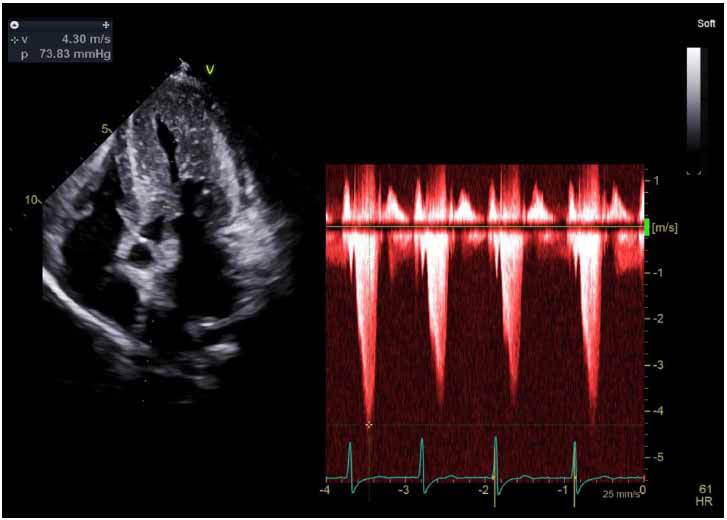
Apical 5-chamber view with simultaneous velocity acquisition through the LVOT for assessment of resting gradient, noted here to be very elevated with a peak of approximately 74 mmHg, meeting diagnostic guidelines criteria for LVOTO.

LVOTO, considered significant if the resting peak gradient is ≥ 30 mm Hg, is present at rest in 30-35% symptomatic HCM patients (Fig. **8**), inducible by physiologic (either bedside maneuvers, like Valsava, or exercise) or pharmacologic provocation in another 30-35% of patients, and not present at rest or with provocation in the remaining 30% [5, 6]. Resting peak LVOT gradients ≥ 30 mm Hg are associated with an increased risk of SCD, and a resting or provocable LVOT gradient ≥ 50 mm Hg is considered the threshold for advanced invasive therapy (such as septal ablation or myomectomy) in HCM patients with symptoms refractory to pharmacotherapy [6, 48].

Multiple physiologic and structural abnormalities contribute to rest and dynamic LVOTO, including changes in LV preload, afterload, and contractility, as well as asymmetric basal septal hypertrophy, and mitral valvular and subvalvular apparatus abnormalities [6, 49]. The large majority of HCM patients with LVOTO have elongated mitral leaflets, both anterior and posterior, that protrude into the LV cavity well above the plane of the mitral annulus, that coapt at the body of the leaflets rather than at the leaflet tips, with the residual portion of the elongated anterior leaflet extending past the displaced coaptation point and contributing to SAM [49, 50]. Hypertrophy, displacement, and bifid morphology of the papillary muscles, particularly the anterolateral papillary muscle, contribute to LVOTO and SAM [6, 49, 51]. The most common pathogenic papillary and chordae abnormalities seen in HCM are antero-basilar displacement of the base of the anterolateral papillary muscle, antero-apical displacement of papillary muscle resulting in chordal-leaflet laxity, the abnormal muscular connection between the anterolateral papillary muscle head and the anterolateral wall into or near A1 MV scallop, bifid morphology, and hypermobile accessory papillary muscles [6, 49, 52, 53]. SAM, while commonly seen in patients with HCM, is not unique to the disease, but contributes to dynamic LVOTO and arises from multiple physical forces, including drag forces on the anterior mitral leaflet and Venturi forces created by flow entering a narrowed LVOT [6, 54].

**Fig. (9):**
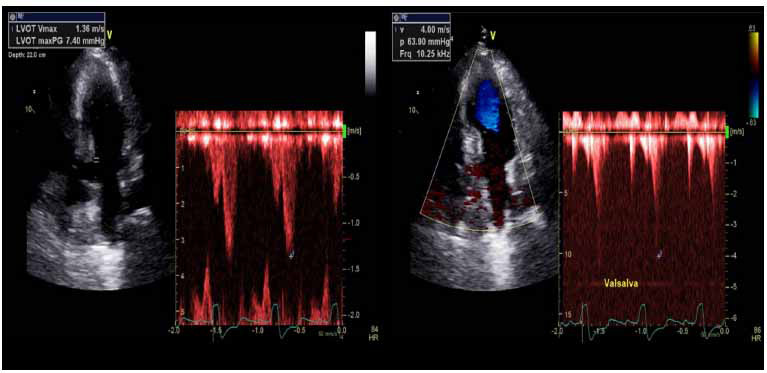
Straining with the Valsava maneuver elicits a sharp rise in LVOT peak gradient, rising from 7.4 mmHg at rest (left) to approximately 64 mmHg (right), meeting diagnostic guidelines criteria for provocable LVOTO.

The dynamic features of LVOTO are dramatically impacted by the loading conditions of preload and afterload, and provocatory maneuvers and challenges, both physiologic and pharmacologic, can be used to assess a provocable LVOT gradient and determine if a patient should be considered for septal reduction therapy [5]. Echocardiography is performed in the post-prandial state, where there is a pooling of blood in the splanchnic circulation from increased parasympathetic tone with resulting decreased central venous and systemic preload, with or without an added fasting stressor with resulting dehydration and a further decrease in preload, may reveal latent LVOTO with an increase in peak LVOT gradient, even in patients HCM patients without post-prandial symptoms [6, 55, 56]. Straining during the Valsava maneuver (Fig. **9**), during which there is forced expiration against a closed airway, increases intrathoracic pressure, decreases systemic venous return and preload, and can precipitate LVOTO [6, 57].

Similarly, going from a squat to standing decreases systemic venous return and preload, thus reducing LV end-diastolic volume and worsening LVOTO [6, 58]. Pharmacologic challenges with amyl nitrite, a systemic arterial vasodilator that decreased afterload with compensatory reflex tachycardia, can also be utilized to illicit latent LVOTO, as well as isoproterenol or dobutamine infusion [3, 6, 59].

While physiologic maneuvers like Valsava straining and squatting to standing are reliable and reproducible reduce venous return and create the loading conditions to precipitate LVOTO, exercise is the most physiologic way to provoke LVOTO [6, 57, 59]. Exercise in the upright position, either on the treadmill or non-recumbent bicycle, provokes a higher gradient than the exercise in the supine position, and although a measured gradient immediately posts upright exercise in the supine or recumbent position may be attenuated when compared to gradients measured in the upright position immediately after or during exercise, technical image acquisition challenges make it reasonable to obtain gradients in the supine position immediately after peak exercise [6, 60].

Many of the same mitral leaflet and apparatus morphologic alterations that predispose the HCM patient to dynamic LVOTO and SAM also contribute to the development and severity of MR. Typical elongation of the anterior MV leaflet relative to the posterior leaflet generates an inter-leaflet gap during systole, resulting in eccentric posteriorly directed MR, with increasing severity related to the degree of leaflet length mismatch and lessened mobility of the posterior leaflet [49, 61]. MR associated with HCM can also arise from intrinsic valvular abnormalities including MV prolapse, leaflet injury from continual septal systolic contact and flow turbulence, and chordal elongation, thickening, and rupture [6].

A comprehensive echocardiographic assessment and quantification of LVOTO, SAM, and MR requires the use of multiple echocardiographic techniques and can involve TTE and TEE, as well as 2D and 3D echocardiography.

**Fig. (10):**
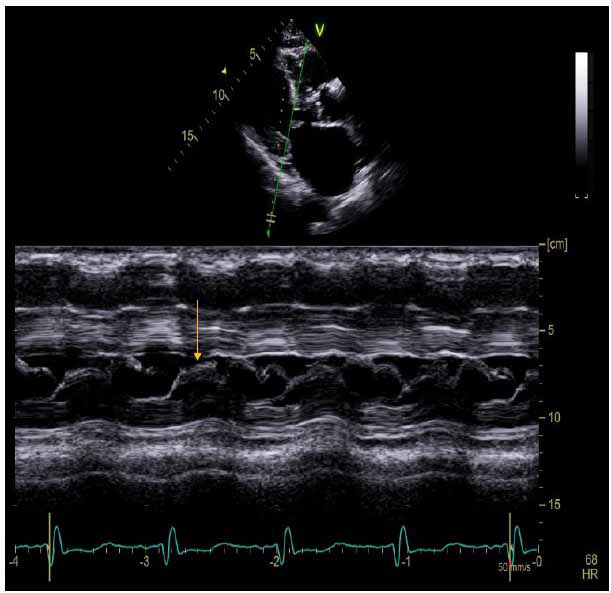
M-mode echocardiographic tracing of the mitral valve in a middle-aged patient with HOCM, revealing the systolic anterior movement of the anterior mitral leaflet (yellow arrow).

M-mode echocardiography, which has been used in the echocardiographic evaluation of HCM since the 1980s and preceded the development of Doppler echocardiography, can be utilized to assess for SAM (Fig. **10**), both the presence and duration [6, 62]. SAM severity has been characterized by SAM-septal distance and duration of leaflet-septal contact, ranging from mild, with SAM-septal distance > 10 mm to severe, with prolonged SAM-septal contact (≥ 30% of systolic duration) [62].

Color flow (CF), Pulsed-wave (PW), and Continuous-wave (CW) Doppler on 2D TTE and TEE, as well as 3D echocardiography, are used to characterize regurgitant flow through the MV and localize turbulent aliased flow corresponding to increased velocity and pressure gradient in the LVOTO or obstruction elsewhere in the LV cavity, as well as the etiology of MR.

**Fig. (11):**
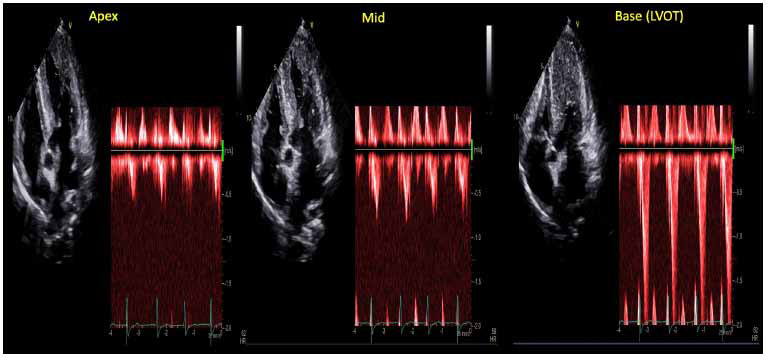
Acquisition of velocities and instantaneous peak gradients at focal points at the LV apex, mid cavity, and LVOT allows for characterization of the level of obstruction. The precipitous rise in velocity and instantaneous peak gradient in the above patient showed obstruction at the level of LVOT, consistent with HOCM.

PW can be used to assess velocities sequentially from the LVOTO all the way down to the LV apex to confirm the anatomical level of the obstruction and should be performed in the apical 5-chamber (A5C) and/or apical 3-chamber views. Determining the peak gradient, reflective of the degree of obstruction, is determined by measuring the peak LVOT velocity using CW Doppler and calculating the gradient with the simplified Bernoulli equation. In addition to determining maximal instantaneous velocities and gradients, CW allows for the characterization of outflow velocity waveforms, with the classical “dagger-shaped” of LVOTO (Fig. **11**) corresponding to the slow increase in velocity during early systole followed by and rapid rise and peaking in mid-to-late systole [6].

**Fig. (12):**
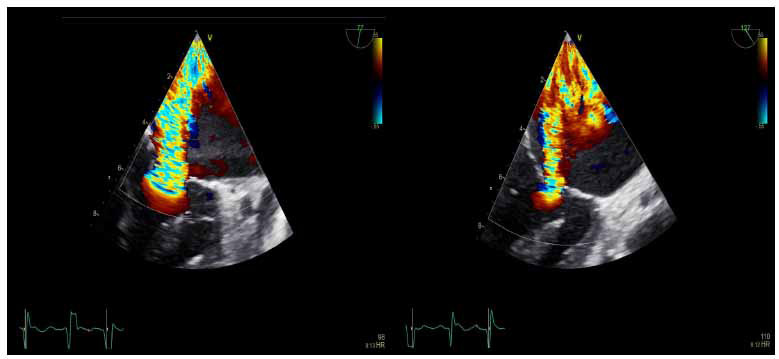
Midesophageal TEE views of the MV to assess for MR, noted to be moderate-severe posteriorly directed MR likely secondary to anterior leaflet lengthening with posterior leaflet asymmetry.

TEE is recommended for patients with poor transthoracic windows and for elucidating the etiology of LVOTO, SAM, and MR [6, 26, 28]. In addition to identifying the mechanism of MR, quantitative assessment (Fig. **12**) of MR severity should be performed, but given the typical eccentricity of MR jets in HCM from leaflet asymmetry, quantification with the proximal isovelocity surface area (PISA) method can result in erroneous estimations. Thus, MR regurgitant volume can be calculated through alternative methods, such as calculating the difference between LV stroke volume (on 2D) and RV stroke volume (systolic right ventricular outflow tract [RVOT] flow determined by multiplying RVOT area by RVOT time velocity integral), assuming there is no significant aortic regurgitation [6].

#### 3.1.4 Echocardiography in Apical and Midventricular HCM

Apical and midventricular HCM morphologic subtypes, with or without midventricular obstruction (MVO), are less common variants but can present with a variety of anatomical substrates and mechanisms causing MVO, including systolic apposition of the hypertrophied mid septum (with hypercontractile LV free wall) or mid-lateral wall (with hypercontractile septum), systolic apposition of hypertrophied papillary muscles, and midventricular narrowing due to middle-to-apical wall hypertrophy with apical aneurysm, with or without apical systolic cavity obliteration [4, 6, 63, 64]. The diagnosis of HOCM from MVO requires a systolic pressure gradient at the midventricular level ≥ 30 mm Hg at rest and LV systolic obliteration unrelated to SAM [6].

It is crucial to distinguish the overarching morphology of the HCM patient found to have an apical aneurysm, and TTE can reveal apical hypertrophy and differentiate between pure and mixed forms of apical HCM [64]. Apical and midventricular HCM, with or without MVO and/or apical aneurysm, are variants of HCM with the use of UEAs on echocardiography can greatly improve diagnosis and identify small apical aneurysms or apical clots that were missed on standard echocardiography [6]. CF, PW, and CW Doppler findings in midventricular and apical HCM can include turbulent flow at the midventricular obstructive level, high CW Doppler velocities persisting through late systole or intracavitary velocities with an abrupt ending, and rapid deceleration, and apical ‘paradoxical’ early diastolic jet corresponding due to the release of high pressure and velocity in the sequestered apical cavity that is released in early diastole [6, 65, 66]. Speckle tracking and strain analysis may demonstrate regional apical dyskinesis in apical HCM, corresponding to cavity obliteration [64].

### 3.2 Cardiac Magnetic Resonance Imaging

CMR, the gold standard for the evaluation of myocardial function, volumes, and tissue characterization, has become an essential component of diagnostic evaluation and risk stratification in HCM [5, 6, 67].

According to the 2020 ACC/AHA guidelines, CMR with gadolinium contrast enhancement should be obtained in the initial imaging evaluation of HCM, not only indicated in patients with suspected or confirmed HCM in whom echocardiography is inconclusive. Furthermore, CMR with contrast enhancement enables more accurate SCD risk stratification with a better assessment of established HCM SCD clinical risk factors. CMR with contrast enhancement should be repeated periodically every 3 to 5 years for serial SCD risk stratification to evaluate for changes in late gadolinium enhancement (LGE) or more frequently depending on baseline and interval measurements of established HCM SCD clinical risk factors, including massive LVH (≥ 30 mm in any segment), and LV apical aneurysm [3, 5].

**Fig. (13):**
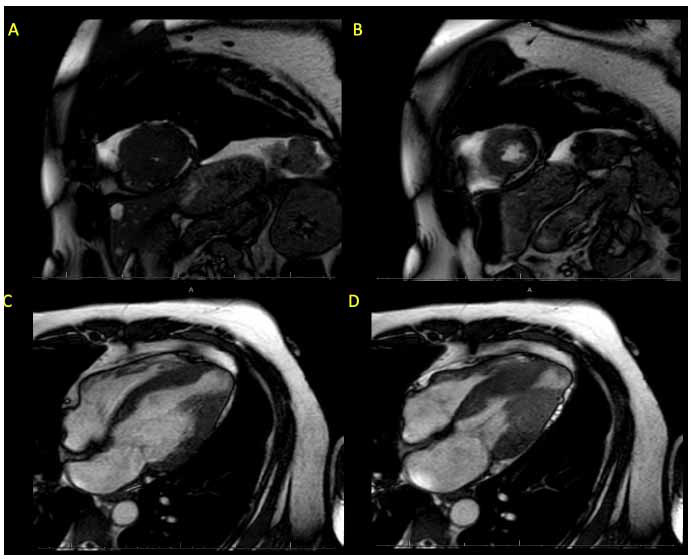
CMR demonstrating mid to apical asymmetric hypertrophy in the patient with mid-apical variant HCM. (**A**) = Short axis at the mid cavity. (**B**) = short axis at the apex. (**C**) = 4 chambers diastolic cine image. (**D**) = 4 chambers systolic cine image.

Multiple features of CMR make it an incredibly valuable complementary modality in the evaluation of suspected or confirmed HCM, including high spatial and temporal resolution, full tomographic imaging of the heart, and quantitative contrast-enhanced assessment of myocardial fibrosis with LGE after gadolinium injection [3, 5, 6]. High resolution enables a sharp definition between the blood pool and endomyocardial borders and thus provides more accurate measurements of wall thickness, particularly septal thickness, by clearly demarcating the borders of adjacent RV muscular structures [3, 5]. Furthermore, the improved spatial and temporal resolution allows for improved visualization and description of phenotypic variants (Fig. **13**), as well as morphologic abnormalities of the mitral leaflets and sub-valvular apparatus, chordae, and papillary muscles [5]. Tomographic imaging enables the identification of hypertrophic in areas of LV that are poorly visualized on standard echocardiographic views, such as the apex and anterolateral free wall [3].

Despite the distinct advantages and additional data that can be obtained from CMR, there are multiple well-recognized limitations to this imaging modality, including higher cost, lack of widespread availability, patient-specific contraindications such as cardiac implantable electronic device MRI incompatibility, chronic kidney disease, and significant claustrophobia [3, 6].

LV systolic function as assessed by LVEF is most accurately measured on CMR and can be determined by CMR in HCM patients with suboptimal echocardiographic views and inconclusive quantitative biplane techniques, despite the use of UEAs [6]. The detailed anatomical assessment of CMR in different planes enables a 3D representation of anatomy with precise contouring of epicardial and endocardial borders for the calculation of quantitative parameters and consistent measurement of atrial and ventricular volumes and function [68]. Bright blood cine imaging with steady-state free precession (SSFP), related to gradient echo imaging, allows for the assessment of global and regional ventricular function and ventricular mass [68, 69]. Strain imaging can also be performed on CMR with techniques known as feature-tracking, tagged-cine, or tissue-tracking, and similar to echocardiography, allows for the assessment of regional wall deformation and strain measurements in the radial, circumferential, and longitudinal directions, reflecting regional and global myocardial systolic function [6, 68, 69]. Abnormalities in global circumferential strain, as well as regional radial and circumferential strain on the CMR, have been identified as predictors of ventricular tachyarrhythmia and appropriate implantable cardioverter-defibrillator discharge, heart failure hospitalization, and mortality [70, 71].

**Fig. (14):**
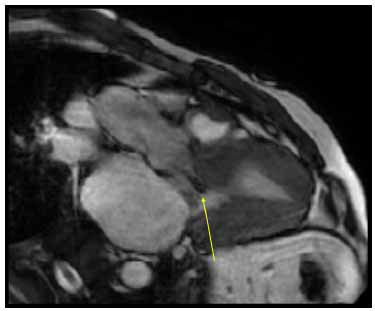
3 chamber systolic cine image in the patient with HOCM showing turbulent flow (black on bright blood imaging) in LVOT (yellow arrow).

For the evaluation of LVOTO and MR, as well as papillary muscle and mitral sub-valvular anatomy, CMR provides better characterization of the anatomy of septal-SAM contact and identifies the mechanisms of LVOTO, as well as isolated or concomitant midventricular obstruction related to midventricular hypertrophy [68, 69]. Multiple CMR sequences enable the comprehensive anatomical characterization of LVOTO (Fig. **14**), including bright blood cine imaging with SSFP and 3D SSFP whole heart dataset sequencing, which allows for more accurate localization of papillary muscle number, extent, and points of proximal and distal attachments [69, 72]. Flow quantification sequences can assess for LVOT flow velocities and profiles, as well as MR regurgitant volumes, with novel techniques such as 4-dimensional flow CMR providing more reproducible techniques for the direct quantification and assessment of MR severity [6, 69, 73].

The full tomographic imaging capabilities of CMR afford it considerable advantages in the evaluation of HCM phenotypic variants [3, 68]. HCM with apical aneurysm, with or without apical hypertrophy, is better assessed by CMR, where aneurysms both large and small missed on TTE can be identified, with post gadolinium contrast imaging further able to delineate the patterns of myocardial scarring and the presence of apical thrombi [6, 69]. In variants of all morphologies, CMR demonstrates a degree of hypertrophy that is not fully appreciated in TTE [74].

The combination of accurate measurements of LVH and direct phenotyping of HCM along with capabilities of tissue characterization using T1, T2, T2* mapping, and LGE collectively highlight the critical role CMR plays in the diagnostic evaluation, monitoring, and serial risk re-stratification of HCM [6, 68].

**Fig. (15):**
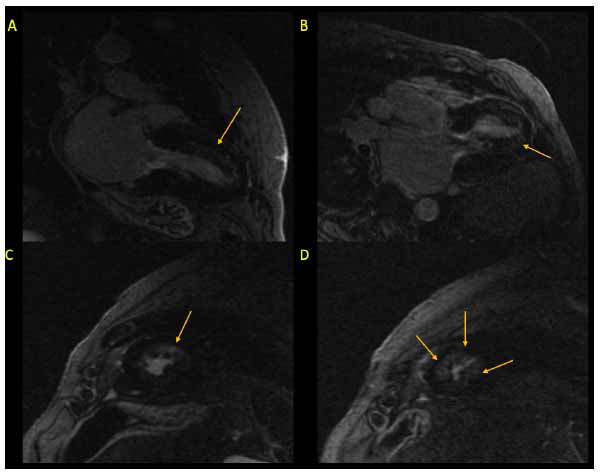
LGE assessment on CMR with gadolinium contrast showing diffuse but patchy LGE (arrows) involving the anteroseptum, anterior, and anterolateral walls extending from mid cavity to apex. (**A**) = 2 Chamber. (**B**) = 3 Chamber. (**C**) = Mid-short axis. (**D**) = Apical short axis.

T1 mapping measurements can assess the total extent of expanded extracellular space and can quantify the extent of subtle and early fibrosis, particularly in patients who are genotype-positive phenotype-negative [68, 75]. The extracellular volume fraction is derived from T1 mapping and measures the proportion of extracellular space between cardiomyocytes, and can detect both regional and diffuse interstitial expansion [6, 68]. The native and post-T1 mapping on a regional and global basis detects patterns of interstitial fibrosis, and T1 relaxation time, which is measured at both the individual myocardial pixel level and regionally, is noted to be increased in correlation with areas of increased wall thickness and LGE [6, 68, 76, 77]. HCM patients with abnormalities of both native T1 values and ECV are at an increased risk of ventricular arrhythmia and SCD [68]. T2 mapping detects interstitial and myocardial water content with increased T2 signal intensity frequently reported in HCM and likely reflective of edema and inflammation, disarray and degeneration of abnormally hypertrophied cardiomyocytes, and replacement fibrosis [6, 68, 78]. Like T1 relaxation time, T2 mapping signals frequently correspond with LGE [68]. T2* blood oxygen level-dependent MRI which evaluates myocardial oxygenation is typically reduced in patients with HCM and corresponds to areas of increased extracellular volume and fibrosis [68, 79]. LGE patterns in HCM (Fig. **15**) vary greatly with a range of location and mural involvement, most commonly affecting the mid-myocardium and correspond with areas of asymmetric hypertrophy outside of a typical epicardial coronary territory [80]. LGE representing myocardial fibrosis found in 65-86% of HCM patients is a well-recognized predictor of SCD and systolic dysfunction in non-HOCM patients and is considered a substrate for diastolic dysfunction with increased myocardial stiffness [68, 80].

### 3.3 Cardiac Computed Tomography

**Fig. (16):**
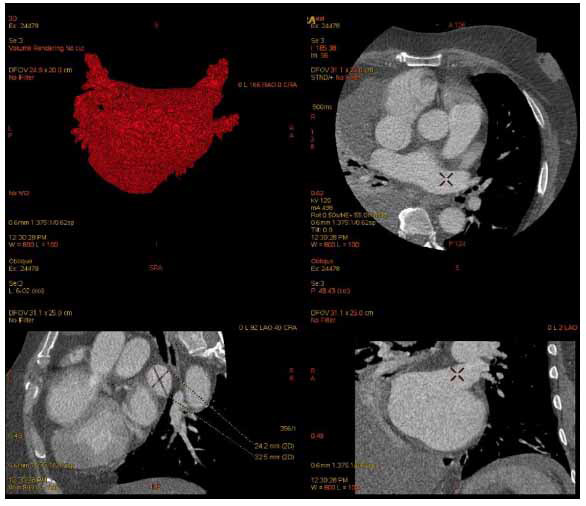
CCT 3D volume rendering of the LA (top left) and mapping of pulmonary vein anatomy and diameter (left superior pulmonary vein, top right and bottom row) prior to AFIB ablation in a 62-year-old male with apical HCM.

CCT has a growing role in the multimodality diagnostic evaluation of HCM, with the 2020 AHA/ACC and 2022 ASE/ASNC/SCMR/SCCT guidelines which indicated that CCT should be considered for patients with nondiagnostic echocardiography and in those in whom CMR is unavailable or cannot be completed due to claustrophobia or noncompatible cardiac implantable electronic device [5, 6]. CCT provides high spatial resolution and enables a clear depiction of morphologic features, including the pattern of hypertrophy, global and regional wall thickness, LV mass, and function, but possesses notable disadvantages of radiation exposure and the use of radioiodine contrast [5]. Furthermore, CCT can reliably detect and quantify myocardial fibrosis [81, 82]. Given the high prevalence of AFIB in HCM, CCT’s well-established role for pulmonary vein mapping (Fig. **16**) prior to AFIB ablation and pulmonary vein isolation enables quantitative analysis of LA size, with LA enlargement and LAVI elevation resulting from the diastolic dysfunction and MR of HCM [3, 83].

For the evaluation of myocardial ischemia and coronary artery disease (CAD) in HCM, CCT and CCT angiography (CCTA) are the premier noninvasive diagnostic imaging modalities with significantly lower false-positive rates in comparison to other noninvasive nuclear cardiology modalities [4, 6]. Myocardial ischemia and angina in HCM arise from multiple potential factors, and frequently are unrelated to obstructive epicardial CAD [6]. Oxygen demand is increased from the higher myocardial mass with increased filling pressures and wall stress, and oxygen supply is diminished due to microvascular dysfunction, intramural coronary arteriole tunica media smooth muscle hypertrophy, myocardial bridging, and reduced myocardial blood flow reserve [6, 84]. Electrocardiogram-synchronized, contrast-enhanced CCTA is useful to assess obstructive epicardial CAD and is considered the reference standard for detecting anomalous coronary artery anatomy and myocardial bridges [6]. While CCT-based fractional flow reserve (CT-FFR) has not been specifically validated in HCM, prior studies have demonstrated that HCM patients have lower cumulative 3-vessel CT-FFR values [6, 85].

### 3.4 Multimodality Imaging and Risk Stratification

Beyond identifying morphologic and physiologic abnormalities, the multimodality diagnostic imaging evaluation of the HCM patient enables prognostication and SCD risk stratification to guide advanced therapies, particularly SCD primary prevention with implantable cardioverter defibrillator (ICD).

The diagnostic advantages and limitations of each modality are summarized in Table **1**.

The 2020 ACC/AHA lists the established clinical risk factors for HCM SCD risk stratification, which include clinical factors like a history of unexplained syncope and family history of SCD from HCM, electrophysiologic factors like non-sustained ventricular tachycardia on ambulatory rhythm monitoring, and diagnostic imaging factors, on either ECHO or CMR, like massive LVH ≥ 30 mm, LV systolic dysfunction with LVEF < 50%, LV apical aneurysm, and extensive LGE [5]. These established risk factors are the parameters used to calculate 5-year SCD risk as devised by the 2014 European Society of Cardiology HCM guidelines, with an ICD recommended for those with a 5-year risk score ≥ 6% (high risk), and intermediate risk categorized as those with a 5-year risk score of 4-6% [86]. According to the 2020 ACC/AHA guidelines, SCD risk assessment should be performed at the initial evaluation and every 1-2 years thereafter [5].

## CONCLUSION

Multimodality imaging, including ECHO, CMR, and CCT is an essential component in the diagnosis, management, and risk stratification in patients with known or suspected HCM, and serial reimaging to assess for progressions in morphologic and physiologic abnormalities are bedrocks of long-term surveillance monitoring. With continued advancements in all these modalities, the knowledge of HCM pathophysiology will simultaneously evolve, as will guidelines and risk stratification strategies.

## Figures and Tables

**Table 1:** Advantages and Disadvantages of Imaging Modalities.

**Modality**	**ECHO**	**CMR**	**CCT**
Advantages	- Noninvasive, non-radiating, portable - Superior in evaluating exercise-induced LVOTO and dynamic MR - UEAs enhance endocardial border visualization for more accurate and reproducible wall and volume measurements that are more closely aligned with CMR measurements	- Noninvasive, non-radiating - Superior image quality in comparison to ECHO - No limitations of imaging windows or planes - Excellent reproducibility and quantification of volumes (including mitral regurgitation), function, mass - LGE – unique tissue characterization for myocardial fibrosis - Accurate characterization of regional deformation (strain) - Quantification and localization of papillary muscles and more accurate anatomical assessment	- Noninvasive detection of obstructive epicardial coronary artery disease - Fractional flow reserve can estimate the severity of intermediate-severity coronary lesions - Volumetric, functional, and mass assessment in patients unable to undergo CMR
Limitations	- Larger patient body habitus and other factors can limit the quality of imaging - TEE - invasive	- Limited availability and portability in comparison to ECHO, Claustrophobia - Unable to perform in patients with non-MRI complaints of cardiac implantable electronic devices - LGE – limited role in patients with chronic kidney disease due to concern for nephrogenic systemic fibrosis - LGE – prolonged acquisition time - LGE – selection of incorrect nulling time may lead to inaccurate measurement of fibrosis - 3D information with the high spatial resolution is more difficult to attain - Accuracy of flow quantification sequences and measurements in HCM not validated	- Similar to CMR, availability, and portability do not match ECHO - Radiation exposure - Nephrotoxic contrast use for CCTA - Electrocardiogram synchronization is required for CCTA, irregular heart rhythm can introduce image artifact

## LIST OF ABBREVIATIONS

HCM: Hypertrophic Cardiomyopathy
LVOT: Left Ventricular Outflow Tract
MR: Mitral Regurgitation
SCD: Sudden Cardiac Death
ECHO: Echocardiography
CMR: Cardiac Magnetic Resonance Imaging
CCT: Cardiac Computed Tomography
AHA/ACC: American Heart Association/American College of Cardiology
ASE/ASNC/SCMR/SCCT: American Society of Echocardiography/American Society for Nuclear Cardiology/Society for Cardiovascular Magnetic Resonance/Society for Cardiovascular Computed Tomography
LV: Left Ventricle
LVH: Left Ventricle Hypertrophy

## References

[1] Liew A., Vassiliou V., Cooper R., Raphael C. (2017). Hypertrophic cardiomyopathy-past, present and future.. J. Clin. Med..

[2] Maron B.J., Maron M.S. (2022). Reflections on six decades of hypertrophic cardiomyopathy from eugene braunwald.. Am. J. Cardiol..

[3] Maron B.J., Desai M.Y., Nishimura R.A. (2022). Diagnosis and evaluation of hypertrophic cardiomyopathy.. J. Am. Coll. Cardiol..

[4] Monda E., Palmiero G., Lioncino M. (2022). Multimodality imaging in cardiomyopathies with hypertrophic phenotypes.. J. Clin. Med..

[5] Ommen S.R., Mital S., Burke M.A. (2020). 2020 AHA/ACC guideline for the diagnosis and treatment of patients with hypertrophic cardiomyopathy.. Circulation.

[6] Nagueh S.F., Phelan D., Abraham T. (2022). Recommendations for multimodality cardiovascular imaging of patients with hypertrophic cardiomyopathy: An update from the American society of echocardiography, in collaboration with the American society of nuclear cardiology, the society for cardiovascular magnetic resonance, and the society of cardiovascular computed tomography.. J. Am. Soc. Echocardiogr..

[7] Maron B.J. (2018). Clinical course and management of hypertrophic cardiomyopathy.. N. Engl. J. Med..

[8] Marian A.J., Braunwald E. (2017). Hypertrophic cardiomyopathy.. Circ. Res..

[9] Neubauer S., Kolm P., Ho C.Y. (2019). HCMR investigators distinct subgroups in hypertrophic cardiomyopathy in the NHLBI HCM registry.. J. Am. Coll. Cardiol..

[10] Hong Y., Su W.W., Li X. (2022). Risk factors of sudden cardiac death in hypertrophic cardiomyopathy.. Curr. Opin. Cardiol..

[11] Cheng Z., Fang T., Huang J., Guo Y., Alam M., Qian H. (2021). Hypertrophic cardiomyopathy: From phenotype and pathogenesis to treatment.. Front. Cardiovasc. Med..

[12] Dai Z., Aoki T., Fukumoto Y., Shimokawa H. (2012). Coronary perivascular fibrosis is associated with impairment of coronary blood flow in patients with non-ischemic heart failure.. J. Cardiol..

[13] Díez J., González A., Kovacic J.C. (2020). Myocardial interstitial fibrosis in nonischemic heart disease, Part 3/4.. J. Am. Coll. Cardiol..

[14] Wijnker P.J.M., Sequeira V., Kuster D.W.D., Velden J. (2019). Hypertrophic cardiomyopathy: A vicious cycle triggered by sarcomere mutations and secondary disease hits.. Antioxid. Redox Signal..

[15] Ommen S.R., Shah P.M., Tajik A.J. (2007). Left ventricular outflow tract obstruction in hypertrophic cardiomyopathy: Past, present and future.. Heart.

[16] Saito C., Minami Y., Haruki S., Arai K., Ashihara K., Hagiwara N. (2022). Prognostic relevance of a score for identifying diastolic dysfunction according to the 2016 American society of echocardiography/European association of cardiovascular imaging recommendations in patients with hypertrophic cardiomyopathy.. J. Am. Soc. Echocardiogr..

[17] Raphael C.E., Cooper R., Parker K.H. (2016). Mechanisms of myocardial ischemia in hypertrophic cardiomyopathy.. J. Am. Coll. Cardiol..

[18] Afonso L.C., Bernal J., Bax J.J., Abraham T.P. (2008). Echocardiography in hypertrophic cardiomyopathy: The role of conventional and emerging technologies.. JACC Cardiovasc. Imaging.

[19] Rakowski H., Carasso S. (2009). Diastolic dysfunction and histopathology in hypertrophic cardiomyopathy: Is relaxation in disarray?. J. Am. Soc. Echocardiogr..

[20] Tower-Rader A., Mohananey D., To A., Lever H.M., Popovic Z.B., Desai M.Y. (2019). Prognostic value of global longitudinal strain in hypertrophic cardiomyopathy.. JACC Cardiovasc. Imaging.

[21] Sheikh N., Papadakis M., Schnell F. (2015). Clinical profile of athletes with hypertrophic cardiomyopathy.. Circ. Cardiovasc. Imaging.

[22] Bos J.M., Towbin J.A., Ackerman M.J. (2009). Diagnostic, prognostic, and therapeutic implications of genetic testing for hypertrophic cardiomyopathy.. J. Am. Coll. Cardiol..

[23] Kim E.K., Lee S.C., Hwang J.W. (2016). Differences in apical and non-apical types of hypertrophic cardiomyopathy: A prospective analysis of clinical, echocardiographic, and cardiac magnetic resonance findings and outcome from 350 patients.. Eur. Heart J. Cardiovasc. Imaging.

[24] Maron M.S., Rowin E.J., Maron B.J. (2017). How to image hypertrophic cardiomyopathy.. Circ. Cardiovasc. Imaging.

[25] Inciardi R.M., Galderisi M., Nistri S., Santoro C., Cicoira M., Rossi A. (2018). Echocardiographic advances in hypertrophic cardiomyopathy: Three-dimensional and strain imaging echocardiography.. Echocardiography.

[26] Venieri E., Aggeli C., Anastasakis A., Sambatakou H., Stefanadis C., Tousoulis D. (2021). Mitral valve in hypertrophic cardiomyopathy: A three-dimensional transesophageal study.. Hellenic J. Cardiol..

[27] Erden M., van Velzen H.G., Menting M.E. (2018). Three-dimensional echocardiography for the assessment of left ventricular geometry and papillary muscle morphology in hypertrophic cardiomyopathy.. J. Ultrasound.

[28] Vainrib A., Massera D., Sherrid M.V. (2021). Three-dimensional imaging and dynamic modeling of systolic anterior motion of the mitral valve.. J. Am. Soc. Echocardiogr..

[29] Lee D.Z.J., Chan R.H., Montazeri M. (2021). Left ventricular apical aneurysms in hypertrophic cardiomyopathy: Equivalent detection by magnetic resonance imaging and contrast echocardiography.. J. Am. Soc. Echocardiogr..

[30] Urbano-Moral J.A., Gonzalez-Gonzalez A.M., Maldonado G. (2020). Contrast-enhanced echocardiographic measurement of left ventricular wall thickness in hypertrophic cardiomyopathy: Comparison with standard echocardiography and cardiac magnetic resonance.. J. Am. Soc. Echocardiogr..

[31] Wang Y., Zhang L., Liu J. (2022). Automated three‐dimensional echocardiographic quantification for left ventricular volume and function in patients with hypertrophic cardiomyopathy.. Echocardiography.

[32] Chen Z., Li C., Li Y. (2021). Layer‐specific strain echocardiography may reflect regional myocardial impairment in patients with hypertrophic cardiomyopathy.. Cardiovasc. Ultrasound.

[33] Serri K., Reant P., Lafitte M. (2006). Global and regional myocardial function quantification by two-dimensional strain: Application in hypertrophic cardiomyopathy.. J. Am. Coll. Cardiol..

[34] van Velzen H.G., Schinkel A.F.L., van Grootel R.W.J. (2019). Five-year prognostic significance of global longitudinal strain in individuals with a hypertrophic cardiomyopathy gene mutation without hypertrophic changes.. Neth. Heart J..

[35] Finocchiaro G., Haddad F., Pavlovic A. (2014). How does morphology impact on diastolic function in hypertrophic cardiomyopathy? A single centre experience.. BMJ Open.

[36] Biagini E., Spirito P., Rocchi G. (2009). Prognostic implications of the Doppler restrictive filling pattern in hypertrophic cardiomyopathy.. Am. J. Cardiol..

[37] Mitter S.S., Shah S.J., Thomas J.D. (2017). A test in context.. J. Am. Coll. Cardiol..

[38] Nagueh S.F., Smiseth O.A., Appleton C.P. (2016). Recommendations for the evaluation of left ventricular diastolic function by echocardiography: An update from the American society of echocardiography and the european association of cardiovascular imaging.. J. Am. Soc. Echocardiogr..

[39] Kitaoka H., Kubo T., Hayashi K. (2013). Tissue doppler imaging and prognosis in asymptomatic or mildly symptomatic patients with hypertrophic cardiomyopathy.. Eur. Heart J. Cardiovasc. Imaging.

[40] Nagueh S.F., McFalls J., Meyer D. (2003). Tissue doppler imaging predicts the development of hypertrophic cardiomyopathy in subjects with subclinical disease.. Circulation.

[41] Yang W.I., Shim C.Y., Kim Y.J. (2009). Left atrial volume index: A predictor of adverse outcome in patients with hypertrophic cardiomyopathy.. J. Am. Soc. Echocardiogr..

[42] Lu D.Y., Haileselassie B., Ventoulis I. (2018). E/e′ ratio and outcome prediction in hypertrophic cardiomyopathy: the influence of outflow tract obstruction.. Eur. Heart J. Cardiovasc. Imaging.

[43] Singh A., Addetia K., Maffessanti F., Mor-Avi V., Lang R.M. (2017). LA strain for categorization of LV diastolic dysfunction.. JACC Cardiovasc. Imag.

[44] Jain V., Ghosh R., Gupta M. (2021). Contemporary narrative review on left atrial strain mechanics in echocardiography: cardiomyopathy, valvular heart disease and beyond.. Cardiovasc. Diagn. Ther..

[45] Lee H.J., Kim H.K., Rhee T.M. (2022). Left atrial reservoir strain-based left ventricular diastolic function grading and incident heart failure in hypertrophic cardiomyopathy.. Circ. Cardiovasc. Imaging.

[46] Rakowski H., Carasso S. (2007). Quantifying diastolic function in hypertrophic cardiomyopathy: The ongoing search for the holy grail.. Circulation.

[47] Chen S., Yuan J., Qiao S., Duan F., Zhang J., Wang H. (2014). Evaluation of left ventricular diastolic function by global strain rate imaging in patients with obstructive hypertrophic cardiomyopathy: A simultaneous speckle tracking echocardiography and cardiac catheterization study.. Echocardiography.

[48] Elliott P.M., Gimeno J.R., Tomé M.T. (2006). Left ventricular outflow tract obstruction and sudden death risk in patients with hypertrophic cardiomyopathy.. Eur. Heart J..

[49] Sherrid M.V., Balaram S., Kim B., Axel L., Swistel D.G. (2016). The mitral valve in obstructive hypertrophic cardiomyopathy.. J. Am. Coll. Cardiol..

[50] Groarke J.D., Galazka P.Z., Cirino A.L. (2018). Intrinsic mitral valve alterations in hypertrophic cardiomyopathy sarcomere mutation carriers.. Eur. Heart J. Cardiovasc. Imaging.

[51] Kwon D.H., Smedira N.G., Thamilarasan M., Lytle B.W., Lever H., Desai M.Y. (2010). Characteristics and surgical outcomes of symptomatic patients with hypertrophic cardiomyopathy with abnormal papillary muscle morphology undergoing papillary muscle reorientation.. J. Thorac. Cardiovasc. Surg..

[52] Teo E.P., Teoh J.G., Hung J. (2015). Mitral valve and papillary muscle abnormalities in hypertrophic obstructive cardiomyopathy.. Curr. Opin. Cardiol..

[53] Kwon D.H., Setser R.M., Thamilarasan M. (2007). Abnormal papillary muscle morphology is independently associated with increased left ventricular outflow tract obstruction in hypertrophic cardiomyopathy.. Heart.

[54] Sherrid M.V., Gunsburg D.Z., Moldenhauer S., Pearle G. (2000). Systolic anterior motion begins at low left ventricular outflow tract velocity in obstructive hypertrophic cardiomyopathy.. J. Am. Coll. Cardiol..

[55] La Canna G., Scarfò I., Arendar I., Alati E., Caso I., Alfieri O. (2020). Phenotyping left ventricular obstruction with postprandial re-test echocardiography in hypertrophic cardiomyopathy.. Am. J. Cardiol..

[56] Adams J.C., Bois J.P., Masaki M. (2015). Postprandial hemodynamics in hypertrophic cardiomyopathy.. Echocardiography.

[57] Jensen M.K., Havndrup O., Pecini R. (2010). Comparison of Valsalva manoeuvre and exercise in echocardiographic evaluation of left ventricular outflow tract obstruction in hypertrophic cardiomyopathy.. Eur. J. Echocardiogr..

[58] Peng L.T., Newman D.B., Geske J.B. (2021). Squat-to-stand provocation of dynamic left ventricular outflow tract obstruction in hypertrophic cardiomyopathy: A case report.. Eur. Heart J. Case Rep..

[59] Ayoub C., Geske J.B., Larsen C.M., Scott C.G., Klarich K.W., Pellikka P.A. (2017). Comparison of valsalva maneuver, amyl nitrite, and exercise echocardiography to demonstrate latent left ventricular outflow obstruction in hypertrophic cardiomyopathy.. Am. J. Cardiol..

[60] Rowin E.J., Maron B.J., Olivotto I., Maron M.S. (2017). Role of exercise testing in hypertrophic cardiomyopathy.. JACC Cardiovasc. Imag.

[61] Schwammenthal E., Nakatani S., He S. (1998). Mechanism of mitral regurgitation in hypertrophic cardiomyopathy: Mismatch of posterior to anterior leaflet length and mobility.. Circulation.

[62] Gilbert B.W., Pollick C., Adelman A.G., Wigle E.D. (1980). Hypertrophic cardiomyopathy: Subclassification by M mode echocardiography.. Am. J. Cardiol..

[63] Elsheshtawy M.O., Mahmoud A.N., Abdelghany M., Suen I.H., Sadiq A., Shani J. (2018). Left ventricular aneurysms in hypertrophic cardiomyopathy with midventricular obstruction: A systematic review of literature.. Pacing Clin. Electrophysiol..

[64] Hughes R.K., Knott K.D., Malcolmson J. (2020). Apical hypertrophic cardiomyopathy: The variant less known.. J. Am. Heart Assoc..

[65] Strachinaru M., Huurman R., Bowen D.J., Schinkel A.F.L., Hirsch A., Michels M. (2022). Relation between early diastolic mid-ventricular flow and elastic forces indicating aneurysm formation in hypertrophic cardiomyopathy.. J. Am. Soc. Echocardiogr..

[66] Po J.R.F., Kim B., Aslam F. (2015). Doppler systolic signal void in hypertrophic cardiomyopathy: Apical aneurysm and severe obstruction without elevated intraventricular velocities.. J. Am. Soc. Echocardiogr..

[67] Salerno M., Sharif B., Arheden H. (2017). Recent advances in cardiovascular magnetic resonance.. Circ. Cardiovasc. Imaging.

[68] Sivalokanathan S. (2022). The role of cardiovascular magnetic resonance imaging in the evaluation of hypertrophic cardiomyopathy.. Diagnostics.

[69] To A.C.Y., Dhillon A., Desai M.Y. (2011). Cardiac magnetic resonance in hypertrophic cardiomyopathy.. JACC Cardiovasc. Imag.

[70] Chen X., Pan J., Shu J. (2022). Prognostic value of regional strain by cardiovascular magnetic resonance feature tracking in hypertrophic cardiomyopathy.. Quant. Imag. Med. Surg..

[71] Pu C., Fei J., Lv S. (2021). Global circumferential strain by cardiac magnetic resonance tissue tracking associated with ventricular arrhythmias in hypertrophic cardiomyopathy patients.. Front. Cardiovasc. Med..

[72] Rajiah P., Fulton N.L., Bolen M. (2019). Magnetic resonance imaging of the papillary muscles of the left ventricle: Normal anatomy, variants, and abnormalities.. Insights Imag.

[73] Gupta A.N., Avery R., Soulat G. (2021). Direct mitral regurgitation quantification in hypertrophic cardiomyopathy using 4D flow CMR jet tracking: Evaluation in comparison to conventional CMR.. J. Cardiovasc. Magn. Reson..

[74] Soler R., Méndez C., Rodríguez E., Barriales R., Ochoa J.P., Monserrat L. (2018). Phenotypes of hypertrophic cardiomyopathy. An illustrative review of MRI findings.. Insights Imag.

[75] Hinojar R., Varma N., Child N. (2015). T1 mapping in discrimination of hypertrophic phenotypes: Hypertensive heart disease and hypertrophic cardiomyopathy.. Circ. Cardiovasc. Imag.

[76] Haaf P., Garg P., Messroghli D.R., Broadbent D.A., Greenwood J.P., Plein S. (2017). Cardiac T1 mapping and extracellular volume (ECV) in clinical practice: A comprehensive review.. J. Cardiovasc. Magn. Reson..

[77] Wong T.C. (2014). Cardiovascular magnetic resonance imaging of myocardial interstitial expansion in hypertrophic cardiomyopathy.. Curr. Cardiovasc. Imaging Rep..

[78] Chen S., Huang L., Zhang Q., Wang J., Chen Y. (2020). T2-weighted cardiac magnetic resonance image and myocardial biomarker in hypertrophic cardiomyopathy.. Medicine.

[79] Gastl M., Gruner C., Labucay K. (2020). Cardiovascular magnetic resonance T2* mapping for the assessment of cardiovascular events in hypertrophic cardiomyopathy.. Open Heart.

[80] Rowin E.J., Maron B.J., Maron M.S. (2020). The hypertrophic cardiomyopathy phenotype viewed through the prism of multimodality imaging.. JACC Cardiovasc. Imag.

[81] Conte E., Mushtaq S., Muscogiuri G. (2021). The potential role of cardiac CT in the evaluation of patients with known or suspected cardiomyopathy: From traditional indications to novel clinical applications.. Front. Cardiovasc. Med..

[82] Langer C., Lutz M., Eden M. (2014). Hypertrophic cardiomyopathy in cardiac CT: A validation study on the detection of intramyocardial fibrosis in consecutive patients.. Int. J. Cardiovasc. Imaging.

[83] Di Cori A., Zucchelli G., Faggioni L. (2021). Role of pre-procedural CT imaging on catheter ablation in patients with atrial fibrillation: Procedural outcomes and radiological exposure.. J. Interv. Card. Electrophysiol..

[84] Basso C., Thiene G., Mackey-Bojack S., Frigo A.C., Corrado D., Maron B.J. (2009). Myocardial bridging, a frequent component of the hypertrophic cardiomyopathy phenotype, lacks systematic association with sudden cardiac death.. Eur. Heart J..

[85] Sellers S.L., Fonte T.A., Grover R. (2018). Hypertrophic cardiomyopathy (HCM): New insights into Coronary artery remodelling and ischemia from FFRCT.. J. Cardiovasc. Comput. Tomogr..

[86] Elliott P.M., Anastasakis A., Borger M.A. (2014). 2014 ESC guidelines on diagnosis and management of hypertrophic cardiomyopathy.. Eur. Heart J..

